# Acute myeloid leukemia and gut microbiome: bidirectional effects and opportunities for intervention

**DOI:** 10.1007/s00277-026-07176-w

**Published:** 2026-07-15

**Authors:** Kirti Arora, Rishi Chowdhary, Aditi Shenoy, Chitresh Kapadia, FNU Paras, Nancy Vora, Aditi Arora, Rahul Chowdhary, Aryan Arora, Manjeet Goyal, Laurie Matt-Amaral

**Affiliations:** 1 Department of Internal Medicine, Cleveland Clinic Akron General, Akron, OH 44307 USA; 2 Department of Internal Medicine, MetroHealth Medical Center, Cleveland, OH USA; 3 Dr. DY Patil Medical College, Navi Mumbai, Maharashtra India; 4 Government Medical College, Miraj, Maharashtra India; 5 Government Medical College, Patiala, Punjab India; 6 Grodno State Medical University, Grodno, Belarus; 7 Punjab Institute of Medical Sciences, Jalandhar, Punjab India; 8 Department of Internal Medicine, Cleveland Clinic, Cleveland, OH USA; 9 University College of Medical Sciences, New Delhi, India

**Keywords:** Gut microbiome, AML, Clonal hematopoiesis, Chemotherapy, Infection, Hematopoietic stem cell transplant, Fecal microbiota transplant

## Abstract

Acute myeloid leukaemia (AML) is characterized by substantial treatment-related morbidity that is not fully explained by cytogenetic or molecular risk stratification alone. Emerging evidence implicates the gut microbiome as a critical, modifiable determinant of AML pathogenesis, therapeutic response, and treatment-related complications. Patients with AML exhibit profound gut microbiome disruption at diagnosis, marked by reduced microbial diversity and depletion of short-chain fatty acid–producing commensals, which is further exacerbated by intensive chemotherapy, antibiotic exposure, and hematopoietic stem cell transplantation (HSCT). These perturbations impair intestinal barrier integrity, amplify systemic inflammation, and promote domination by opportunistic and multidrug-resistant organisms, thereby increasing the risks of mucositis, bloodstream infections, graft-versus-host disease, and mortality. Beyond complications, microbiome-derived metabolites modulate immune signalling, hematopoietic homeostasis, and inflammatory pathways relevant to clonal haematopoiesis and leukemogenesis, suggesting bidirectional interactions between the microbiome and AML biology. Observational and translational studies demonstrate that preserved microbial diversity and enrichment of specific taxa are associated with improved survival following HSCT, while dysbiosis correlates with inferior outcomes. Interventions aimed at microbiome preservation and restoration—including antibiotic stewardship, dietary modulation, autologous or donor fecal microbiota transplantation, and emerging postbiotic strategies—have shown feasibility and biologic efficacy, although definitive clinical benefit remains to be established. This review synthesizes current mechanistic, clinical, and translational evidence linking gut microbiome dysregulation with AML outcomes, highlights emerging therapeutic strategies, and outlines key challenges and future directions. Integrating microbiome-focused supportive care into AML management represents a promising avenue to reduce treatment toxicity and improve patient outcomes.

## Introduction

The therapeutic landscape of acute myeloid leukemia (AML) has transformed substantially over the past decade. Traditional induction and consolidation regimens have been augmented by maintenance strategies and molecularly targeted therapies- such as FLT3 and IDH inhibitors- and by venetoclax-based combinations that extend options across distinct genomic subgroups [1]. Despite these advances, AML continues to carry high treatment-related morbidity and disappointing long-term survival, particularly among older adults and those with adverse-risk mutations [1].

Cytogenetic and molecular profiling have refined prognostication, enabling risk-based treatment selection and measurable residual disease (MRD)-guided strategies [1]. Yet, these tools do not fully capture inter-individual variability in outcomes such as infection susceptibility, immune reconstitution, or chemotherapy tolerance. Emerging data suggest that host-related factors, particularly the gut microbiome, may help explain this variability and influence both therapeutic response and complications [1].

The gut microbiome- a dynamic ecosystem of bacteria, fungi, viruses, and archaea- exerts systemic immunometabolic effects through production of metabolites such as short-chain fatty acids (SCFAs). These compounds regulate epithelial integrity, modulate cytokine networks, and calibrate innate and adaptive immunity [2]. Clinical studies now show that AML patients exhibit marked microbiome disruption at diagnosis, progressive loss of diversity during intensive chemotherapy, and metabolomic alterations associated with inflammation, anorexia, and sarcopenia [2–4]. Interventions such as autologous fecal microbiota transfer (AFMT) or donor fecal transplantation have shown potential to restore microbial diversity following chemotherapy [2, 5].

Collectively, these findings position the gut microbiome as a modifiable determinant of treatment outcomes in AML—one that bridges oncologic biology, host immunity, and therapeutic tolerance. This review synthesizes current evidence linking microbiome dysregulation with AML pathogenesis, therapy response, and complications, and discusses translational avenues for microbiome-based interventions.

## Gut microbiome

The human gastrointestinal tract harbors one of the densest microbial ecosystems known, with the colon alone containing approximately 10^11 organisms per milliliter [6]. The community comprises bacteria, fungi, viruses, and archaea; among them, bacteria predominate both numerically and functionally. Two major phyla—Firmicutes and Bacteroidetes—constitute nearly 90% of bacterial taxa, followed by smaller contributions from Actinobacteria, Proteobacteria, and Verrucomicrobia [7]. Commensal fungi such as *Candida* species and diverse bacteriophages coexist in symbiosis with bacterial communities, maintaining mucosal and immunologic balance [8, 9].

Gut microbes interact intimately with the host immune system. Through pattern-recognition signaling, microbial antigens shape dendritic-cell and T-cell differentiation, fostering tolerance toward commensals while enabling rapid pathogen defense [10]. Commensal-derived SCFAs, notably butyrate and propionate, promote regulatory T-cell maturation, suppress NF-κB–driven inflammation, and enhance intestinal barrier integrity [11]. Microbes also reinforce epithelial barriers, reducing translocation of bacterial products such as lipopolysaccharide (LPS) [12]. Disruption of these functions—as occurs during AML therapy—impairs mucosal defenses, heightens systemic inflammation, and predisposes to microbial translocation [3, 13].

Beyond local intestinal effects, microbial metabolites act along multiple organ axes. Through the gut-liver axis, dysbiosis alters bile-acid metabolism and contributes to hepatic inflammation and metabolism-associated steatotic liver disease [14].Reduced butyrate-producing taxa impair insulin sensitivity and promote type 2 diabetes [15], while microbial metabolites such as trimethylamine-N-oxide (TMAO) have been implicated in atherosclerosis and cardiovascular mortality [15, 16]. Microbiome perturbations are also integral to autoimmune and inflammatory disorders—loss of *Faecalibacterium prausnitzii* is characteristic of inflammatory bowel disease, whereas *Prevotella copri* expansion correlates with rheumatoid arthritis [17, 18]. In oncology, the enrichment of *Fusobacterium nucleatum* in colorectal cancer underscores the microbiome’s capacity to influence carcinogenesis and tumor immunity [19, 20].

These diverse immunometabolic roles provide a biologic rationale for exploring microbiome involvement in AML. Dysbiosis may influence leukemogenesis through chronic inflammation and altered hematopoietic signaling, affect chemotherapy metabolism and mucosal integrity, and modulate transplant-related immune outcomes. Understanding this interplay is crucial for integrating microbiome preservation into AML care.

## Pathophysiology in AML

### Clonal hematopoiesis

Clonal hematopoiesis of indeterminate potential (CHIP) represents an age-associated expansion of hematopoietic stem-cell clones carrying mutations commonly implicated in leukemia- such as *DNMT3A*, *TET2*, and *ASXL1*- in the absence of overt malignancy [21]. Chronic inflammation is recognized as a key driver of CHIP progression, and recent work has implicated the gut microbiome as a modifiable contributor to this inflammatory milieu [10, 21].

**Fig. 1:**
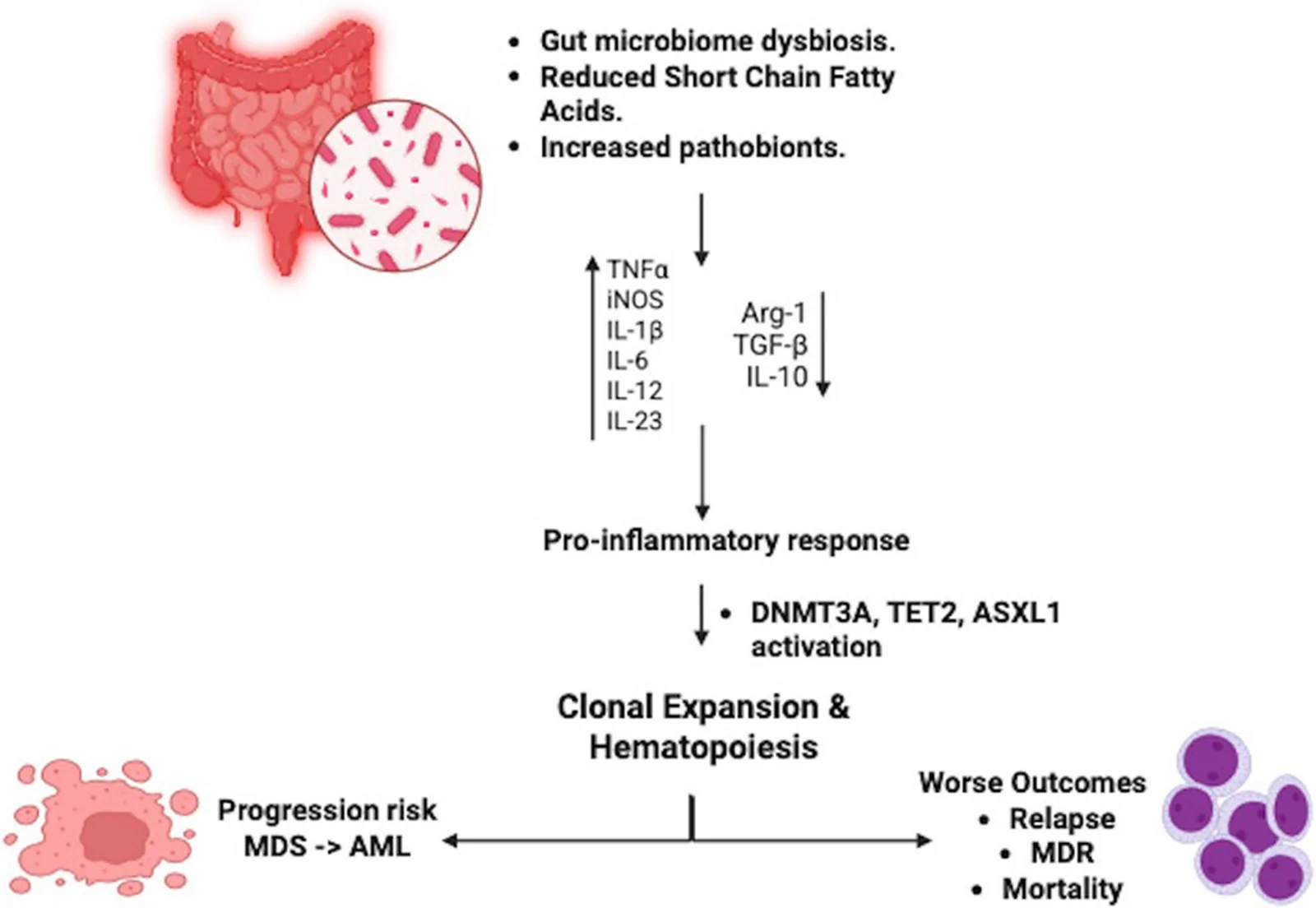
Gut microbiome dysbiosis perpetuated clonal hematopoiesis and chronic inflammation

Antibiotic-induced depletion of commensal bacteria in murine models leads to pronounced hematopoietic changes, including impaired myeloid differentiation and bone-marrow hypocellularity [22]. More recently, *Agarwal et al.* (2025) demonstrated that a bacterial metabolite, ADP-heptose, derived from Gram-negative organisms, can activate NF-κB signaling and promote the expansion of *DNMT3A*-mutant clones [22]. This metabolite was detectable in the circulation of older adults and associated with increased systemic inflammation and cardiovascular events. These findings collectively identify the gut microbiota as an upstream, reversible driver of inflammatory signaling and clonal hematopoiesis, suggesting that microbiome modulation could potentially mitigate leukemogenic risk (Fig. 1).

### Microbiome alteration at diagnosis and post-therapy

Patients with AML exhibit substantial perturbations in gut microbiome composition even before initiation of therapy. Multiple studies have demonstrated reduced overall microbial diversity at diagnosis, with depletion of beneficial SCFA-producing genera- *Faecalibacterium*, *Roseburia*, *Lacticaseibacillus*, and *Collinsella-* and concomitant enrichment of opportunistic taxa such as *Enterococcus* [23]. These shifts likely reflect chronic inflammation, disease-related anorexia, and prior antibiotic exposure. Functionally, this dysbiosis correlates with systemic inflammation, sarcopenia, and impaired metabolic resilience, factors that can worsen chemotherapy tolerance [23].

During induction chemotherapy, intestinal barrier injury and broad-spectrum antibiotic use exacerbate dysbiosis. Obligately anaerobic commensals nearly vanish, while *Enterococcus*, *Klebsiella*, and *Escherichia* species proliferate. Although partial microbial recovery occurs over ensuing weeks, longitudinal studies reveal that many patients fail to regain baseline diversity, retaining a pro-inflammatory, low-diversity microbiome months after hematologic recovery [23].

**Fig. 2:**
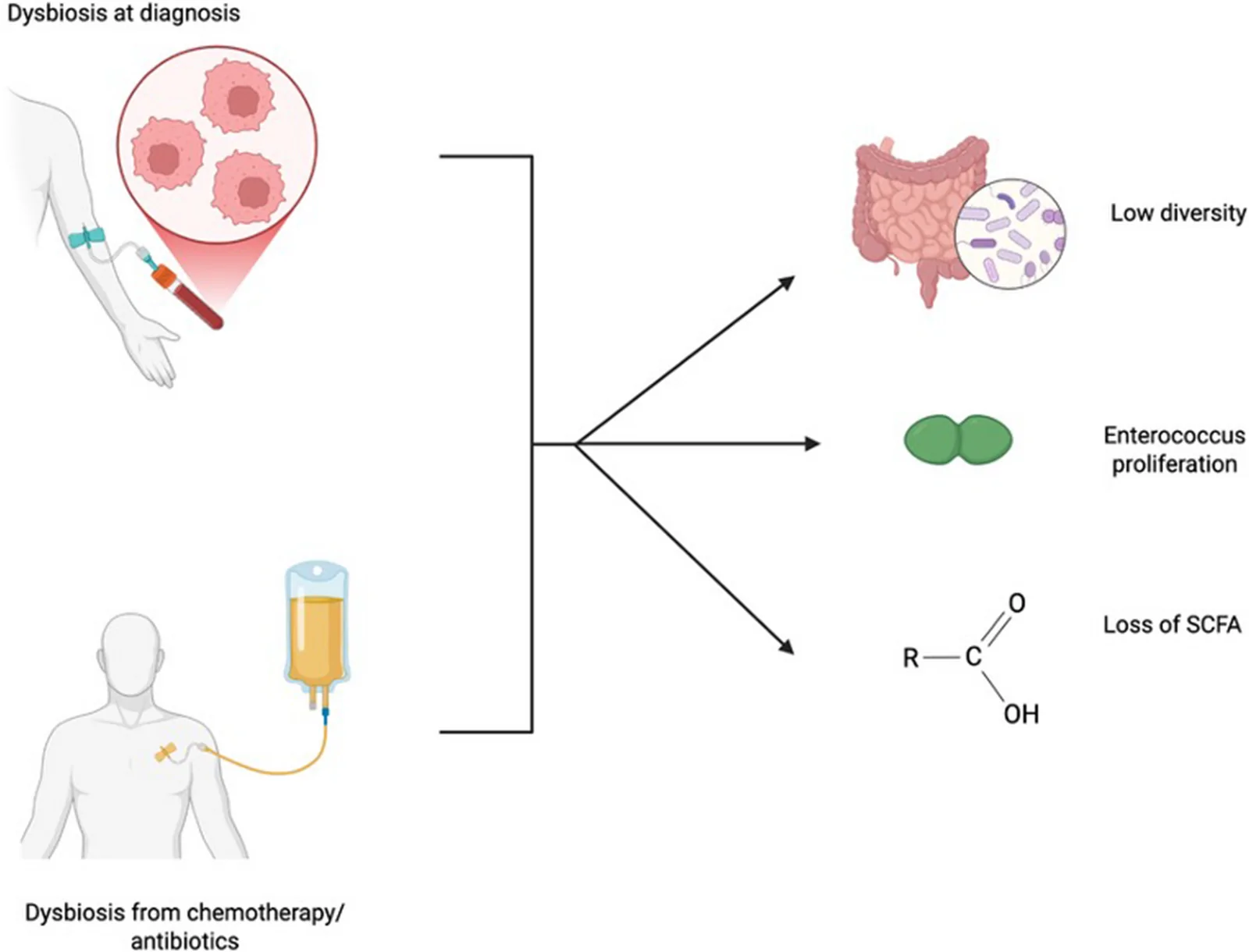
Pathophysiology of microbiome alteration in AML

Importantly, these microbial shifts are not mere bystanders. Higher baseline *Enterococcus* abundance and loss of *Faecalibacterium* correlate with elevated bone-marrow blast percentages and systemic inflammatory markers [23] (Fig. 2). Dysbiosis may thereby serve as both a reflection and a driver of leukemia biology, influencing cytokine networks, intestinal permeability, and metabolic signaling. Thus, AML and its treatment together create a bidirectional feedback loop in which leukemia-associated inflammation promotes dysbiosis, and dysbiosis, in turn, may foster disease progression and post-therapy complications.

Another question that arises is whether specific AML-defining mutations are associated with distinct microbiome patterns. Current evidence suggests that no mutation-specific gut microbiome patterns have been identified in AML. This hypothesis was tested by Salvestrini et al., and they found no significant differences in the gut microbiota composition when patients were stratified by FLT3, NPM1, IDH1/2, or TP53 mutational status at diagnosis [24]. Other studies examining the AML gut microbiome at diagnosis have similarly not stratified or identified mutation-specific patterns [23, 25]. Future studies will be essential to determine whether mutation-specific inflammatory programs leave a detectable imprint on the gut microbiome, and whether such patterns could serve as biomarkers or therapeutic targets.

## Artificial sweeteners and gut microbiome- a common household ingredient with deleterious effects?

Diet profoundly shapes the gut microbiome, influencing both its composition and metabolic output. Artificial sweeteners, widely used as sugar substitutes, are a pertinent example. Even seemingly minor dietary additives can induce measurable microbial shifts with systemic effects. Non-nutritive sweeteners (NNS) - saccharin, sucralose, aspartame, and stevia- are particularly relevant because they are ubiquitous in modern diets and often consumed by patients attempting to maintain caloric control during therapy.

Experimental studies show that NNS can modify gut bacterial communities and alter glucose metabolism, immune signaling, and inflammatory tone [26]. Sucralose and saccharin have been linked to reduced microbial diversity, enrichment of pro-inflammatory taxa, and impaired glucose tolerance, whereas stevia appears to exert a milder or even prebiotic effect, enhancing the growth of SCFA-producing commensals [26]. However, individual responses to artificial sweeteners can vary depending on the person’s baseline microbiome composition, and dose and duration of sweetener exposure are critical factors [26].

Recent translational work extends these findings into oncology. Morder at al. demonstrated that the consumption of sucralose in mice and humans can impair T-cell–mediated anti-tumor immunity and blunt the efficacy of immune checkpoint inhibitors by disrupting gut microbial balance [27]. Although AML therapy does not currently rely on checkpoint blockade, these data highlight a broader principle: dietary constituents can modulate systemic immunity through microbiome-dependent pathways.

For AML patients- many of whom experience taste changes, mucositis or loss of appetite during treatment, such evidence warrants caution. “Sugar-free” alternatives, while attractive, might inadvertently aggravate dysbiosis or inflammatory stress. Incorporating dietary counseling into AML care—focusing not only on caloric adequacy but also on preservation of microbial diversity—could therefore become an important adjunct to support the health of the gut microbiome.

## Impact on treatment outcomes

### Response and toxicity to chemotherapy

Although AML therapy outcomes have traditionally been attributed to cytogenetic and molecular risk, accumulating evidence suggests that gut microbial composition at baseline and during treatment also influences therapeutic response and toxicity. Most studies to date focus on treatment-related complications- particularly mucosal injury and infection- yet several mechanistic insights hint at a broader impact on chemotherapy efficacy itself.

Importantly, not all chemotherapy regimens have equivalent effects on the gut microbiome. Renga et al. demonstrated that CPX-351, unlike conventional 7 + 3 combination, protected against gut dysbiosis and mucosal damage through activation of the aryl hydrocarbon receptor (AhR)–IL-22–IL-10 host pathway and promotion of immunomodulatory metabolites by anaerobic commensals [28]. This finding was further supported by real-world data demonstrating that CPX-351 is associated with significantly fewer severe enteral infections than conventional chemotherapy [29]. These findings suggest that the liposomal formulation may spare the intestinal epithelium, preserving microbial ecology and colonization resistance. Whether other AML-directed therapies, such as venetoclax-based combinations, FLT3 inhibitors, and IDH inhibitors, have similarly distinct microbiome signatures remains an important area for future investigation.

The mechanisms underlying these regimen-specific differences likely relate to the degree of intestinal barrier injury and subsequent microbial disruption. Loss of commensal diversity during induction chemotherapy leads to intestinal barrier breakdown, endotoxemia, and chronic inflammation [3]. This inflammatory milieu not only exacerbates mucositis and febrile neutropenia but may also alter drug metabolism and immune-mediated tumor surveillance [30]. The gut microbiota participates in xenobiotic metabolism, modulating bile-acid pools and enzymatic activation of chemotherapeutic agents [31]. Dysbiosis could therefore influence drug bioavailability or cytotoxicity, a concept well recognized in solid-tumor models but only recently explored in AML.

Preclinical data further support the functional relevance of these interactions. Animal studies illustrate that specific commensals can enhance responsiveness to cytotoxic chemotherapy and immunostimulatory agents by shaping tumor-associated immune microenvironments [30]. Conversely, antibiotic-induced depletion of these taxa attenuates anti-tumor responses and increases regimen-related toxicity [31, 32]. Translating these findings to AML, where chemotherapy and emerging immunotherapies intersect, suggests that preserving microbial diversity might augment both treatment tolerance and anti-leukemic efficacy. While human data remain preliminary, this avenue holds promise for individualized microbiome-based supportive strategies.

### Response to hematopoietic stem cell transplant

Allogeneic hematopoietic stem cell transplantation (HSCT) is a curative therapy for many high-risk AML patients, and the peri-transplant gut microbiome has emerged as a powerful, independent determinant of post-transplant outcomes. Intensive conditioning regimens and prophylactic antibiotics induce profound dysbiosis characterized by loss of obligate anaerobes and expansion of pathobionts such as Enterococcus [33].

In a landmark study by Peled et al., patients with high gut microbial diversity at engraftment demonstrated significantly better overall survival and lower graft-versus-host disease (GVHD)–related mortality [13]. The presence of protective taxa such as Blautia (a butyrate-producing commensal) was associated with reduced GVHD severity, while Enterococcus dominance correlated with inferior survival [34, 35]. The study by Theoringer et al., which looked at mouse and human microbiomes, reported worse outcomes with enterococcus expansion post HSCT [36]. Mechanistically, mucosal injury during conditioning impairs lactose digestion, creating a nutrient niche that promotes Enterococcus overgrowth and exacerbates GVHD—an effect mitigated in preclinical models by lactose restriction [36]. Intriguingly, specific commensals may also influence relapse risk. Eubacterium limosum abundance post-HSCT was linked to lower leukemia relapse rates, suggesting that microbial metabolites might modulate graft-versus-leukemia effects [37].

Together, these findings indicate that maintaining a balanced microbiota—through antibiotic stewardship, dietary modulation, or microbiome restoration—could improve transplant success by reducing GVHD and infectious mortality while preserving anti-leukemic immunity.

## Impact on treatment related complications

### GI complications

The gastrointestinal (GI) tract is a major target of collateral damage during AML treatment. Chemotherapy drugs, especially in high doses, attack the rapidly dividing cells of the intestinal epithelium, leading to mucositis and increased intestinal permeability. The gut microbiome plays an integral role in modulating these toxicities. Patients who develop severe oral/gut mucositis during induction tend to have lower gut microbial diversity compared to those with minimal mucositis and subsequently mucosal injury- associated blood stream infections [38].

After allogeneic transplant, GI complications are dominated by acute graft-versus-host disease of the gut. Again, the microbiome’s role is evident: lower gut microbial diversity around engraftment is associated with a higher likelihood of developing severe GI GVHD [39]. Patients who go on to develop gut GVHD frequently show an early loss of many SCFA producers. The lack of SCFA production (especially butyrate) removes a crucial anti-inflammatory signal for intestinal immune cells, potentially facilitating the unchecked donor T-cell activation that characterizes GVHD [40].

### Infectious complications

Infectious morbidity remains a major challenge in AML therapy, closely intertwined with the state of the gut microbiome. Under normal circumstances, a diverse commensal ecosystem confers colonization resistance, limiting the growth and translocation of pathogenic organisms. Chemotherapy and broad-spectrum antibiotics disrupt this balance, allowing opportunists such as Enterococcus faecium, Klebsiella, and Escherichia to dominate [41] .

Gut domination by vancomycin-resistant Enterococcus (VRE) is a well-validated predictor of subsequent bloodstream infection (BSI) and sepsis [41]. Similarly, prolonged carbapenem exposure (> 72 h) during induction markedly reduces microbial diversity and increases infection risk four-fold in the ensuing 90 days [42]. Beyond enterococci, overgrowth of *Klebsiella*,* Escherichia*, and *Enterobacter* often occurs in neutropenic patients on prolonged antibiotics. These organisms can carry numerous antibiotic resistance genes and are a major cause of sepsis in AML [38]. Evidence suggests that gut microbiota affect infectious outcomes even after the acute neutropenic phase. In the study by Peled et al., the diversity of the gut microbiome at 30 days post-HSCT was inversely correlated with the risk of late infections and even relapse [13]. This also significantly affects overall survival [13, 35].

The link extends to fungal infections: antibacterial prophylaxis with fluoroquinolones can favor fungal overgrowth and candidemia, illustrating the delicate ecological balance between bacterial and fungal communities [43].

**Fig. 3:**
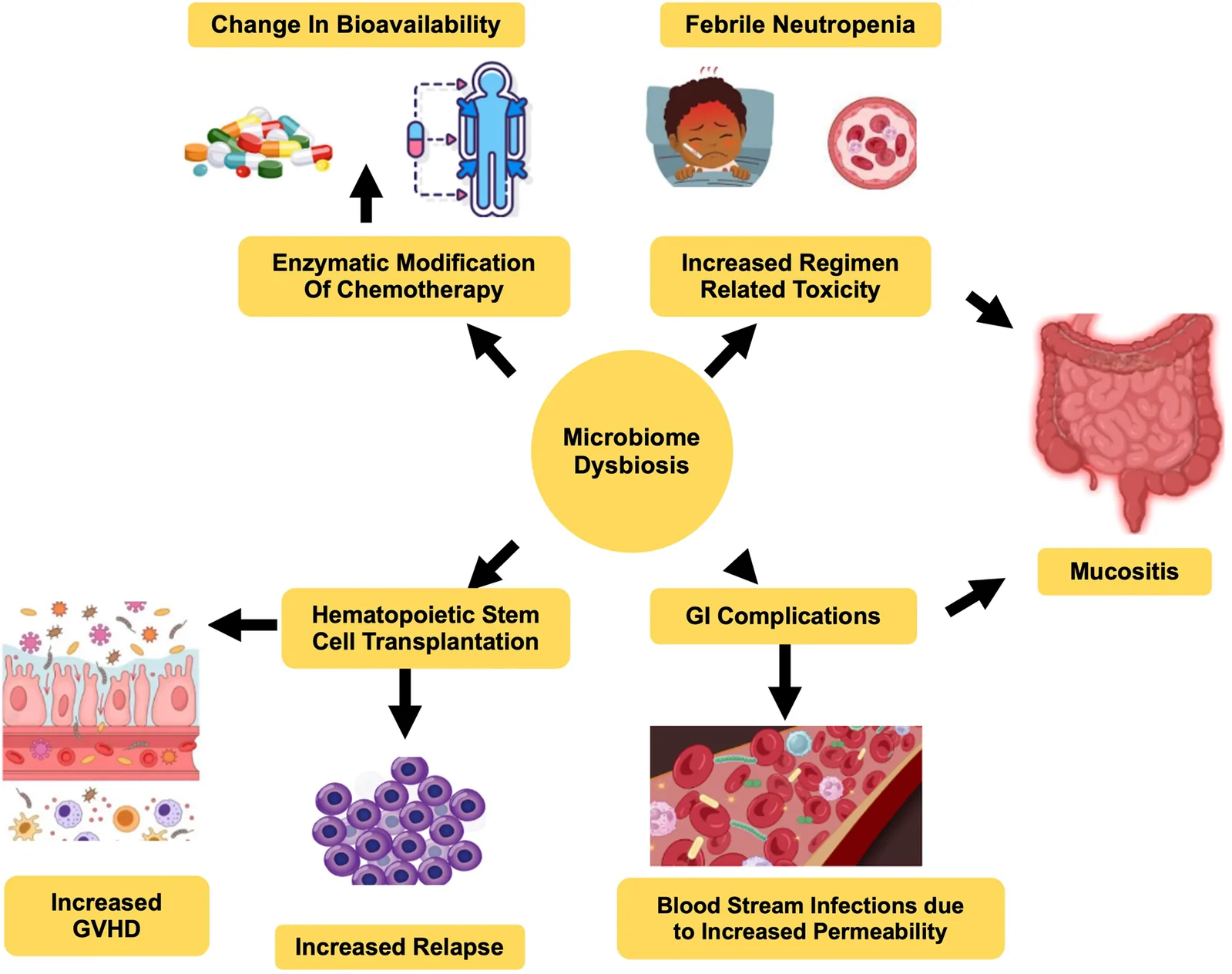
Adverse effects of gut microbiome dysbiosis in patients with AML

The degree of microbial diversity itself appears prognostic. Patients with a high baseline Shannon diversity index at the start of induction are substantially less likely to develop infections during the neutropenic phase, whereas diversity < 2 strongly predicts infectious complications [42]. Collectively, these findings underscore the microbiome as both a biomarker and mediator of infection risk, suggesting that strategies aimed at preserving commensal diversity may improve supportive care outcomes in AML (Fig. 3).

## Gut microbiome preserving strategies

### Antibiotic stewardship

Antibiotic use is a double-edged sword in AML care. On one hand, prompt empiric antibiotics for febrile neutropenia are lifesaving and standard-of-care. On the other hand, broad-spectrum antibiotics (especially those with anti-anaerobic activity) are the primary drivers of microbiome devastation during therapy. A careful balance must be struck. Guideline-concordant antibacterial prophylaxis in AML hinges on anticipated depth/duration of neutropenia. ASCO/IDSA recommend antibiotic prophylaxis with fluoroquinolone and antifungal prophylaxis with an oral triazole or parenteral echinocandin for patients at high risk (ANC < 100/µL for > 7 days) [44]. ECIL-10 adds to the stewardship: narrow empiric therapy, avoid routine anti-anaerobic coverage, and discontinue antibiotics once patients are clinically stable and afebrile ≥ 48 h - even if neutropenia persists [45]. As our ability to monitor the microbiome improves, future strategies might include real-time microbiome sequencing to guide antibiotic choices. The goal is to prevent lethal infections while minimizing collateral damage to the microbial allies that protect the patient.

### Fecal microbiota transplant

Fecal microbiota transplantation has garnered significant interest in the context of AML. The rationale is straightforward: if dysbiosis contributes to infections and other complications, then correcting the dysbiosis should confer benefit.

Autologous FMT (auto-FMT) has the advantage of eliminating the risk of transmitting infectious or undesirable organisms from another person. A pilot study by Malard et al. (2021) demonstrated the feasibility of auto-FMT in AML patients after intensive chemotherapy. Patients who had banked their stool prior to induction received it back once neutropenia resolved. The procedure was safe and led to a restoration of gut microbial diversity close to baseline levels. Importantly, auto-FMT recipients showed a reduction in antibiotic-resistant organisms in their stool, suggesting that reintroduction of their native microbiota may help displace hospital-acquired resistant strains [5].

A randomized phase II trial by Rashidi et al. evaluated an oral encapsulated donor FMT product versus placebo in patients either undergoing allogeneic transplant or receiving intensive chemotherapy for AML [46]. The results showed that FMT was safe and effectively increased gut bacterial diversity compared to placebo [46]. However, the primary endpoint- reduction in severe infections- was not significantly different between the FMT and placebo groups in that trial [46].

In the phase 1b CIMON trial (2025), an oral pooled-donor microbiota product (MaaT033) was given to 21 AML patients during aplastic periods after chemotherapy. The therapy was well tolerated and led to sustained increases in commensal diversity and butyrate-producing bacteria. There were also signals of reduced inflammatory markers in the blood and a trend toward better 2-year survival, though the study was not powered for clinical endpoints [47].

Overall, FMT-based interventions represent a promising strategy to accelerate microbiome recovery in AML patients. Restoring a balanced microbiome could theoretically reduce infections (by re-establishing colonization resistance), decrease GI toxicity (by boosting mucosal healing and anti-inflammatory metabolites), and even enhance anti-leukemia immunity. The challenge is proving these benefits in randomized trials. As of now, FMT is not a standard of care for leukemia patients, but it is offered in controlled trial settings. If ongoing trials demonstrate tangible clinical improvements, FMT or microbiome therapy could become a routine adjunct in AML treatment, akin to growth factor support or antiviral prophylaxis.

### Nutritional and probiotic supplementation

Another avenue to support the gut microbiome in AML is through diet and supplements. During intensive therapy, many AML patients struggle with oral intake due to mucositis, nausea, and taste changes. Ensuring adequate nutrition is itself vital for immune function and healing. Beyond basic nutritional needs, there is interest in diets or supplements that could favorably shift the microbiome.

In immunocompetent populations and some cancer cohorts, probiotics have been shown to reduce antibiotic-associated diarrhea and even mitigate chemotherapy-related GI symptoms [48]. However, larger meta-analyses have yielded mixed results, with some failing to show a statistically significant benefit of probiotics in preventing chemotherapy-associated diarrhea across multiple trials [49]. This inconsistency means the efficacy of probiotics is not yet definitive. More importantly, there have been case reports of probiotic organisms causing bloodstream infections in neutropenic patients, of *Lactobacillus* sepsis in a patient on probiotic therapy and numerous others [50–52]. Although rare, this risk has led major guidelines to advise against routine probiotic use in profoundly immunosuppressed patients outside of clinical trials.

Nonetheless, the concept of manipulating the microbiome with benign microbes remains attractive. To maximize safety, some researchers are focusing on non-live interventions, such as postbiotics, metabolites produced by bacteria, like butyrate [53, 54]. While these are non-AML and largely preclinical, they provide a mechanistic rationale to study postbiotics in myelosuppressed patients. In the near future, we may see tailored microbiome nutritional plans for AML patients, potentially including prescription postbiotics, once their safety and efficacy are proven in this vulnerable population (Table 1).

**Table 1:** Summary of interventions to mitigate gut dysbiosis

Intervention	Outcome	Mechanism	Key studies	Limitations
Antibiotic stewardship [45]	Reduce dysbiosis, preserve diversity, decrease VRE/MDRO domination and downstream infections	Minimize collateral damage to commensals; preserve colonization resistance; prevent VRE domination	ECIL-10 guidelines recommending narrow empiric therapy and early discontinuation	Must balance infection control with stewardship
Autologous FMT post chemotherapy [5]	Restore pre‑treatment microbiome, increase diversity, reduce antibiotic‑resistant organisms	Re‑introduction of patient’s banked stool re‑establishes native commensals and colonization resistance	Malard et al. (auto-FMT) showing diversity restoration	Requires pre‑induction stool banking; Resource‑intense; trial‑limited; long‑term effects unknown
Donor FMT / oral microbiota products [46]	Accelerate microbiome recovery, possibly reduce inflammation and complications	Transplant of screened donor consortia or pooled products reseeds depleted anaerobes and butyrate producerS	Rashidi et al. and CIMON trials (donor FMT) showing increased diversity and safety	Not yet standard of care, Regulatory constraint, Trial limited
Probiotics [48]	Mitigate chemotherapy related diarrhea, support barrier function	Introduce selected commensals to modulate local inflammation and epithelial integrity	Mixed RCT/meta‑analysis data in oncology	Rare but serious risk of bloodstream infection (Lactobacillus, Saccharomyces fungemia); Major guidelines discourage routine use in profound neutropenia: Limited AML‑specific evidence
Postbiotics [53]	Enhance mucosal healing, reduce inflammation without live organisms	Directly delivers the key anti-inflammatory and barrier-repairing metabolite (Butyrate), bypassing the need for a live microbe	Preclinical and non-AML studies demonstrating anti-inflammatory effects and gut barrier enhancement	Mostly non‑AML and early phase; Optimal dosing, formulation, and safety in cytopenic patients not established
Lactose restriction [36]	Limit Enterococcus expansion and GI GVHD severity	Reduces luminal lactose that fuels Enterococcus overgrowth after conditioning‑induced lactase loss	Stein‑Thoeringer Lactose drives Enterococcus expansion to promote GVHD.	Human interventional data limited; dietary adherence and nutritional adequacy need monitoring
Dietary counseling [27]	Preserve microbial diversity and SCFA production; avoid NNS‑linked immune dysfunction	Reduces sucralose/saccharin exposure that perturbs microbiota; promotes fermentable fiber for SCFA generation	Preclinical work on sucralose blunting anti-tumor immunity	Supportive Care (Increasingly recognized as important, but not a standardized “microbiome diet”).

## Challenges and future direction

While the interplay between the gut microbiome and AML outcomes is increasingly evident, there are several challenges and open questions on the path to clinical integration. First, the heterogeneity of patient microbiomes and the dynamic changes during treatment make it difficult to pinpoint universal “good” vs. “bad” microbiome profiles. Patient-specific factors like age, diet, prior antibiotic exposures, and co-morbidities shape the gut flora and thus a strategy that benefits one patient might not have the same effect on another. Large, longitudinal studies are needed to map the microbiome trajectories of AML patients and identify which features are consistently prognostic or actionable.

Second, intervening in the microbiome must be done carefully in immunosuppressed hosts. As noted, probiotics carry a rare but serious risk of infections [50]. FMT, while generally well tolerated, could transmit undetected pathogens or genes. Despite rigorous donor screening, there is always a theoretical concern of something like an asymptomatic virus or a bacterium with resistance genes transferring to the recipient. The long-term consequences of intentionally altering someone’s microbiome are also unknown - could it inadvertently increase risk of other diseases? These concerns mean that any microbiome-based therapy will require thorough testing in clinical trials with safety as a top priority. Regulatory standards are still evolving for these therapies; for example, FMT is currently regulated in many countries as a biologic drug, which necessitates pharmaceutical-grade manufacturing if it’s to be widely used.

Another challenge is determining the optimal timing and context for microbiome intervention. Should we aim to prevent dysbiosis e.g. by giving probiotics or FMT prophylactically at the start of induction? Or is it better to rescue the microbiome after the acute insult e.g. FMT at neutrophil recovery? Preventative strategies might maintain gut integrity and prevent complications, but they run the risk of interfering with necessary treatments. Rescue strategies fit more naturally after intensive therapy, but by then some damage is done. Ongoing trials are exploring multiple windows – some give FMT right after antibiotics, others wait until just before discharge from hospital, etc., to see when it confers the most benefit [46].

From a research perspective, multi-omics approaches will be crucial in future studies. Metabolomics (profiling microbial and host metabolites in blood and stool) can complement microbiome sequencing to provide a functional readout of what the microbiome is actually doing. This could reveal, for example, that two patients with different microbiome compositions might have similar metabolite profiles, or vice versa, which could better explain differences in outcomes. The concept of a “functional microbiome” signature (as opposed to simply listing bacterial species) may be more relevant biologically. For instance, identifying that an AML patient’s stool lacks butyrate and propionate may be more actionable (suggesting need for SCFA supplementation) than knowing what bacterium that patient lacks per se, because one could replace the function (SCFA) even if the exact microbe is not restored.

In terms of clinical impact, one of the most immediate applications of microbiome research in AML could be risk stratification. Just as we profile a patient’s cytogenetics, we might profile their gut microbiome at diagnosis to identify those at higher risk of complications. A patient with extremely low diversity and a preponderance of multidrug-resistant organisms in their gut might benefit from enhanced monitoring, early antifungal prophylaxis, or even consideration of less intensive therapy if the risk of infectious death is deemed too high with standard induction.

Additionally, this vision of microbiome-based risk stratification will require validated clinical tools for monitoring. Several methods are available for microbiome profiling, including 16 S rRNA amplicon sequencing and shotgun metagenomic sequencing, both endorsed by the 2025 International Consensus Statement on Microbiome Testing [55]. These methods can quantify alpha diversity (e.g., Shannon index), beta diversity, and taxonomic composition. In AML, a baseline Shannon diversity index < 2 has been proposed as a predictor of infectious complications during induction, and emerging frameworks advocate for baseline ecosystem phenotyping incorporating metagenomic and metabolomic data [42, 56]. However, no microbiome monitoring tool has been validated for routine clinical decision-making in AML. Key barriers include lack of standardized protocols, absence of validated clinical thresholds, turnaround time, cost, and the need for matched healthy control comparisons. As sequencing costs decrease and bioinformatic pipelines become more accessible, integration of microbiome monitoring into AML risk stratification and supportive care represents a promising but still investigational frontier.

Finally, integrating microbiome care into AML management will require cultural shifts in supportive care practices. Finding the balance between necessary antimicrobial use and microbiome preservation is tricky. It will involve educating healthcare providers about the microbiome’s role, developing new clinical guidelines that explicitly address microbiome health, and potentially involving dietitians and microbiologists as part of the leukemia care team.

In conclusion, the gut microbiome represents both a challenge and an opportunity in AML. The challenge is that modern treatments inevitably damage this organ, leading to complications. The opportunity is that unlike human genetics or leukemia mutations, the microbiome can be changed. As we move forward, a multidisciplinary effort combining clinical trials, microbial genomics, and patient-centered care will be essential to harness the microbiome for improving AML outcomes. The ultimate vision is an integrated approach to leukemia therapy - one that eradicates the malignant cells while nurturing the patient’s microbiome, thereby optimizing the chances of cure with minimal collateral harm.

## Data Availability

No datasets were generated or analysed during the current study.
